# International representation of authors, editors and research in neurology journals

**DOI:** 10.1186/s12874-021-01250-9

**Published:** 2021-03-22

**Authors:** Teodora Bojanic, Aidan Christopher Tan

**Affiliations:** 1 Faculty of Medicine, University of New South Wales, Sydney, New South Wales Australia; 2 School of Medicine, Western Sydney University, Sydney, New South Wales Australia; 3 South Western Sydney Clinical School, University of New South Wales, Sydney, New South Wales Australia

**Keywords:** International, Representation, Authors, Editors, Neurology, Journals

## Abstract

**Background:**

Published research informs international healthcare, yet only a few studies have assessed the representation of authors, editors, and research from developing countries in biomedical journals.

**Methods:**

We reviewed all research articles published in five high-ranking peer-reviewed neurology journals (*The Lancet Neurology*, *Acta Neuropathologica, Nature Reviews Neurology, Brain* and *Annals of Neurology*) in 2010 and 2019 to determine the extent of contributions of authors, editors and research from developing countries, and the degree of international research collaboration between developed and developing countries.

**Results:**

First authorship was attributed to authors from developing countries in only 2% (11/729) of research articles in 2010 and 3% (19/647) of research articles in 2019. All 144 editorial board members in 2019 were from developed countries. International research collaboration between developing and developed countries accounted for only 4% (30/729) of all research articles in 2010 and 6% (40/647) of all research articles in 2019.

**Conclusions:**

There is urgent need for strategies to support high-quality and contextually appropriate biomedical research in developing countries. Supporting high quality and contextually appropriate biomedical research now is necessary for developing countries to meet the rising healthcare needs of their populations in the future.

**Supplementary Information:**

The online version contains supplementary material available at 10.1186/s12874-021-01250-9.

## Highlights

Published research should reflect the diversity of global health.

Representation of authors, editors and research from developing countries is rare in high-ranking neurology journals, and this has not improved over the past decade.

There is an urgent need for strategies to support high quality and contextually appropriate biomedical research in developing countries.

International research organisations can contribute to reducing the disparity in research through multinational research collaborations, and international peer-reviewed journals can contribute to improving the diversity of authors through writing support services and to improving the diversity of editors through international advisory boards. Supporting high quality and contextually appropriate biomedical research now is necessary for developing countries to meet the rising healthcare needs of their populations in the future.

## Introduction

### Background

Biomedical and public health research underpins evidence-based medical practice and global health policy. The evidence which informs practice and policy often originates from research articles published in high-impact peer-reviewed scientific journals. However, several studies [1–4] have reported that developing countries, as defined by a low human development index (HDI), are underrepresented in the first authors of these research articles and editorial board members of these scientific journals. Very low representation of first authors from developing countries has been reported in orthopaedics (1), psychiatry (2), pharmacology (3), and general medicine (4). Authors from developing countries contributed to only 6.5% of clinical research literature published in five high-impact general medical journals in 2004 [4]. A similarly low representation of editorial board members from developing countries has been observed in leading peer-reviewed tropical medicine journals [5]. Several obstacles to research from developing countries have been proposed, including English language proficiency, limited research funding and technical support, and editorial discretion to publish content relevant to the primary readership [1, 3, 4].

Published research should reflect the diversity of global healthcare and the impact that social, cultural and geographical factors can have on local health practice and policy. Yet no studies have examined the contribution of developing countries to authors, editors or research in neurology, nor have any studies assessed collective authorship (as opposed to first authorship) or international collaborations in any biomedical discipline. The need for research in neurology cannot be understated. Globally, neurological disorders are the leading cause of disability and second leading cause of death [6]. Low and middle-income countries bear almost 80% of the burden of neurological disorders, and this is only projected to rise as the rapidly ageing population grows [7].

### Objectives

The aim of this study was to determine the representation of authors, editors and research from developing countries in high ranking peer-reviewed neurology journals and the degree of international research collaboration between developed and developing countries.

## Methods

We conducted a cross-sectional study of all research articles published in 2010 and 2019 in the five highest ranked peer-reviewed neurology journals. Journals were eligible for inclusion if they were peer-reviewed, their primary scope was neurology, and they were established prior to 2010. Journals were ranked by the 2018 SCImago Journal Rank indicator, a measure of the mean number of citations received by articles published in a journal in the preceding 3 years. The included journals were *The Lancet Neurology, Acta Neuropathologica*, *Nature Reviews Neurology*, *Brain*, and *Annals of Neurology*.

All research articles published in the included journals in 2010 and 2019 were electronically retrieved using the electronic bibliographic databases MEDLINE via Ovid and EMBASE via Ovid and manually checked against the websites of included journals. Research articles eligible for inclusion included systematic reviews, meta-analyses, reviews, randomized controlled trials, quasi-experimental studies, cohort studies, case-control studies, cross-sectional studies, case reports, experimental studies (e.g., research involving immunohistochemistry, biogenetics, imaging or the analysis of human brain tissue) and animal-based studies.

We reviewed the full-text publication of all included articles and extracted information on the article title, publication year, publication journal, study design, number of references, citation count, number of authors, each author’s national affiliation, and origin of data. Each author’s national affiliation was defined as the country listed in the institutional affiliation of the author. Where two institutional affiliations with different countries were listed for an author, the country listed in the first institutional affiliation was taken as the author’s national affiliation. For included articles with more than 100 authors, we reviewed the contributions section and only included authors involved in either the study design, statistical analysis or original draft.

For each included article, each author’s national affiliation was classified as developed or developing according to the 2019 Human Development Report [8]. Collective authorship national affiliation was defined by the proportion of authors per research article whose country of national affiliation was a developing or developed country. If all authors were from developing countries, collective authorship was attributed to a developing country. Conversely, if all authors were from developed countries, collective authorship was attributed to a developed country. Where collective authorship was evenly split between developing and developed countries, collective authorship was attributed to a developing country if the first author was from a developing country or to a developed country if the first author was from a developed country. Origin of data was defined as the country which contributed the greatest number to the sample size. If a multinational study did not specify the sample size for different countries, origin of data was classified as international.

The citation count for each included article was retrieved from Scopus, Elsevier’s abstract and citation database (which includes self-citations), between January 5–6, 2020 and June 9–10, 2020, for the 2010 and 2019 articles, respectively.

We reviewed each included journal and extracted information on the citation rates and impact factor values for 2010 and 2019, number of editorial board members in 2019, and geographical demographic of the editorial boards. The national affiliation of editorial board members was defined in the same way as the national affiliation of authors.

Article, author and journal characteristics were analyzed descriptively with frequencies and proportions (percentage). Differences in both collective authorship and first authorship in developed and developing countries between 2010 and 2019 were analyzed statistically with Pearson’s chi-squared tests with Yates correction.

## Results

We identified 1800 articles published in 2010 and 1188 articles published in 2019. After excluding duplicates and non-research articles, we included 729 articles published in 2010 and 647 articles published in 2019. The distribution of study designs was similar between research articles published in 2010 and 2019. The majority of research articles were experimental studies, contributing 53% in 2010 (*n* = 383) and 51% in 2019 (*n* = 327). Review articles contributed 19% of research articles in 2010 (*n* = 142) and 18% of research articles in 2019 (*n* = 119). The remaining study designs (i.e., randomized controlled trials, cohort studies, case reports, systematic review and/or meta-analysis, case control studies, and cross-sectional studies) contributed 27% of research articles in 2010 (*n* = 204) and 32% of research articles in 2019 (*n* = 201).

### Collective authorship

In research articles published in 2010, collective authorship was attributed to developing countries in 2% (*n* = 11) and developed countries in 98% (*n* = 718) (Table 1). In research articles published in 2019, collective authorship was attributed to developing countries in 3% (*n* = 20) and developed countries in 97% (*n* = 627). International research collaborations between developing and developed countries accounted for 4% (30/729) of all research articles in 2010 and 6% (40/647) of all research articles in 2019.

**Table 1:** Collective authorship (developed vs. developing)

	2010 (*n* = 729)	2019 (*n* = 647)
Developing	11 (2%)	20 (3%)
Developed	718 (98%)	627 (97%)

A Pearson’s chi-squared test with Yates correction examining the relationship between collective authorship in developed and developing countries in 2010 and 2019 was not significant [X^2^ (1, *N* = 1376) = 3.2116 *p* = .073], indicating no change in collective authorship in developed and developing countries between 2010 and 2019.

Table 2 summarizes collective authorship attributed to developing and developed countries across the included journals. Overall, all included journals had a very low proportion of research articles with authors from developing countries. The only notable change between 2010 and 2019 was a 4% relative increase in the number of research articles from authors in developing countries in the *Annals of Neurology*.

**Table 2:** Collective authorship by journal

	2010 (*n* = 729)	2019 (*n* = 647)
**Acta Neuropathologica**		
Developing	1 (1%)	1 (1%)
Developed	125 (99%)	128 (99%)
**Annals of Neurology**		
Developing	0 (0%)	7 (4%)
Developed	187 (100%)	186 (96%)
**Brain**		
Developing	7 (3%)	7 (4%)
Developed	267 (97%)	192 (96%)
**Nature Reviews Neurology**		
Developing	0 (0%)	1 (2%)
Developed	52 (100%)	40 (98%)
**The Lancet Neurology**		
Developing	3 (3%)	4 (5%)
Developed	87 (97%)	81 (95%)

Across both 2010 and 2019, collective authorship attributed to developing countries contributed significantly fewer research articles across all study designs compared to those attributed to developed countries (Table 3).

**Table 3:** Collective authorship by type of study

	2010 (*n* = 729)	2019 (*n* = 647)
**Developing**		
Experimental Study	5 (45%)	7 (35%)
Animal Study	3 (27%)	4 (20%)
Review Articles	0 (0%)	4 (20%)
Randomized Controlled Trials	1 (9%)	3 (15%)
Case Reports	0 (0%)	1 (5%)
Case Control Studies	0 (0%)	1 (5%)
Cohort Studies	1 (9%)	0 (0%)
Cross Sectional Study	1 (9%)	0 (0%)
Systematic Reviews and/or Meta-Analysis	0 (0%)	0 (0%)
Quasi Experiments	0 (0%)	0 (0%)
**Developed**		
Experimental Study	378 (53%)	320 (51%)
Review Articles	142 (20%)	115 (18%)
Animal Study	87 (12%)	58 (9%)
Randomized Controlled Trials	44 (6%)	48 (8%)
Cohort Studies	28 (4%)	41 (7%)
Case Reports	24 (3%)	8 (1%)
Systematic Reviews and/or Meta-Analysis	3 (0%)	23 (4%)
Case Control Studies	8 (1%)	10 (2%)
Cross Sectional Study	4 (1%)	4 (1%)
Quasi Experiments	0 (0%)	0 (0%)

### First authorship

In research articles published in 2010, first authorship was attributed to developing countries in 2% (*n* = 11) and developed countries in 98% (*n* = 718) (Table 4). In research articles published in 2019, first authorship was attributed to developing countries in 3% (*n* = 19) and developed countries in 97% (*n* = 628).

**Table 4:** First authorship (developed vs. developing)

	2010 (*n* = 729)	2019 (*n* = 647)
Developing	11 (2%)	19 (3%)
Developed	718 (98%)	628 (97%)

Table S1 summarizes the number of first authors by national affiliation. First authorship was primarily attributed to authors from developed countries including the United States of America, United Kingdom, Germany, Netherlands, and France, and there was no notable change between 2010 and 2019. China, classified as a developing country, was the national affiliation for 8 first authors in 2010 and 17 first authors in 2019.

A Pearson’s chi-squared test with Yates correction examining the relationship between first authorship from developed and developing countries in 2010 and 2019 was not significant [X^2^ (1, *N* = 1376) = 3.2762 *p* = .070], indicating no change in first authorship from developed and developing countries from 2010 to 2019.

### Research origin

Table S2 denotes the primary origin of data for the research articles in the included journals for the same periods. In both 2010 and 2019, data primarily originated from United States of America, followed by international datasets, United Kingdom, and Germany. Of the 729 publications in 2010, only 16 (2.2%) included data from a developing country, and of the 647 research articles in 2019, only 21 (3.2%) included data from a developing country.

### Editorial board membership

We identified 144 members of editorial boards from the included journals in 2019. All editorial board members were from developed countries.

## Discussion

### Key results

Collective authorship and first authorship from developing countries is rare in research articles published in high-ranking peer-reviewed neurology journals and has not improved over the past decade. This furthers the findings of very low representation of authors from developing countries reported in orthopaedics [1], psychiatry [2], pharmacology [3], and general medicine [4], and provides evidence that this phenomenon is not isolated to first authorship, but extends to collective authorship. Although we found a small absolute increase in the number of first authors affiliated with a developing country (from 11 authors in 2010 to 19 authors in 2019), this increase stemmed predominantly from China. This underrepresentation of developing countries in authorship extended to editorial board membership, with no editorial board members from a developing country across all included journals, and international research collaborations, with infrequent research collaboration between developing and developed countries.

The quantity and quality of research conducted by investigators from developing countries or conducted in developing countries is likely impaired by poor funding and facilities, lack of government incentives, and inadequate training and support [3, 4]. These barriers likely contribute to a scientific environment and research conditions which fall short of competitive peer-reviewed journal standards. Certainly, journals are entitled to publish research that is of interest to their readership, for instance, cross-institutional clinical trials and experimental research; this is particularly true of the five high-ranking journals we reviewed. However, the agenda setting role of these journals means that they should support, where possible, the dissemination of neurology research relating to the developing world. Journals could support this endeavor and improve the diversity of editors by establishing international advisory boards.

While there are occasional collaborations between developed and developing countries, these, too, are infrequent. Nevertheless, there is a general trend towards multinational research collaboration groups. We found that cross-institutional collaborations were broadly attributed to the ‘West’ (i.e., United States of America, United Kingdom, Australia etc.), ‘Europe’ (i.e., France, Germany, Switzerland etc.), and ‘Scandinavia’ (Netherlands, Sweden, Denmark, Finland etc.). Interestingly, although countries from Asia, namely Japan and China, contributed to about 5% of research articles published in the included journals in 2019, there was minimal cross-institutional collaboration. Although these trends in multinational research collaborations may reflect geographical conveniences and international relationships, they may be inadvertently perpetuating the neglected representation of developing nations in research.

### Limitations

This study focused on the five highest ranked peer-reviewed neurology journals and, because English is the lingua franca of biomedical research, all five journals only published articles in the English language. Therefore, the results of this study may not be generalizable to peer-reviewed neurology journals which are lower ranked or publish articles in other languages. This study only assessed research articles which were published and, therefore, cannot differentiate the degree to research articles by authors from developing countries were not published because they were not submitted by the author or were rejected by the journal. Nevertheless, this is the first study to assess collective authorship (rather than only first authorship) and international research collaboration between developing and developed countries across any biomedical discipline.

### Generalizability

There is an urgent need for strategies to support high-quality medical research in developing countries. There are wide-ranging benefits to supporting research in developing countries. Local researchers in developing countries benefit from exposure to greater research opportunities, education, and training. This is beneficial to developing countries as they are able to direct socially and culturally relevant research that is readily applicable to local healthcare systems. National and international research bodies are well placed to reduce this disparity in healthcare research through cross-institutional and multinational collaborations which foster long-term mutual learning opportunities and strengthen the quality of research in developing countries [1].

Meeting the neurological needs of those in developing countries remains a pressing issue. Although developing countries have the greatest burden of neurological disease [6], they have fewer neurologists per capita [9, 10]. Despite many countries sending graduates abroad for training due to lack of comprehensive teaching in their own countries, many graduates do not return home to practice [11]. For those who do return, they may find themselves ill-equipped to manage the diseases endemic to their home nation or unable to utilize the limited resources for medical practice [9]. Cross-institutional research collaborations offer an opportunity to bridge this educational gap by cultivating knowledge in diseases which are endemic to developing regions, while simultaneously strengthening the quality of research in developing countries.

Although English is not the primary language in most developing countries, rejection based solely on language is undesirable. Influential international journals should be encouraged to offer free checking and editing services for manuscripts from non-English speaking scientists following their acceptance for publication [3]. While the submission guidelines for the selected journals recommended the use of professional English language editing services if required, the suggested services were all operated externally by for-profit companies. None of the five journals offered free editing services or fee waivers for developing countries.

Many of these strategies present logistical and economical barriers, and as such, serious thought must be put into how these are to be implemented. These are only the first steps to ensuring we lessen the global research disparity and improve the quality and impact of international healthcare research.

## Supplementary Information

**Additional file 1 Table S1.** First authorship (country).

**Additional file 2 Table S2.** Origin of data.

## Data Availability

The datasets generated and analysed during the current study, and the descriptive analyses, are available in the Open Science Framework repository, https://osf.io/am5hk/?view_only=21a00c6dfda344999b6957b9a0b6572e.

## References

[1] Aluede EE, Phillips J, Bleyer J, Jergesen HE, Coughlin R (2012). Representation of developing countries in orthopaedic journals: a survey of four influential orthopaedic journals general. Clin Orthop Relat Res.

[2] Patel V, Sumathipala A (2001). International representation in psychiatric literature: Survey of six leading journals. Br J Psychiatry.

[3] Rohra DK (2011). Representation of less-developed countries in pharmacology journals: an online survey of corresponding authors. BMC Med Res Methodol.

[4] Sumathipala A, Siribaddana S, Patel V (2004). Under-representation of developing countries in the research literature: Ethical issues arising from a survey of five leading medical journals. BMC Med Ethics.

[5] Keiser J, Utzinger J, Tanner M, Singer BH (2004). Representation of authors and editors from countries with different human development indexes in the leading literature on tropical medicine: survey of current evidence. BMJ..

[6] Feigin VL, Nichols E, Alam T, Bannick MS, Beghi E, Blake N, et al. Global, regional, and national burden of neurological disorders, 1990–2016: a systematic analysis for the global burden of disease study 2016. Lancet Neurol. 2019;18(5):459–80. 10.1016/S1474-4422(18)30499-X. 10.1016/S1474-4422(18)30499-X PMC6459001 30879893

[7] Feigin VL, Vos T, Nichols E, Owolabi MO, Carroll WM, Dichgans M, Deuschl G, Parmar P, Brainin M, Murray C (2020). The global burden of neurological disorders: translating evidence into policy. Lancet Neurol.

[8] UNDP. 2019. Human Development Report 2019. Beyond income, beyond averages, beyond today: Inequalities in human development in the 21st century. New York. http://hdr.undp.org/en/content/human-development-report-2019.

[9] Bergen DC (2002). Training and distribution of neurologists worldwide. J Neurol Sci.

[10] World Health Organization (2004). Atlas: country resources for neurological disorders 2004.

[11] Stilwell B, Diallo K, Zurn P, Vujicic M, Adams O, Dal PM (2004). Migration of health-care workers from developing countries: strategic approaches to its management. Bull World Health Organ.

