# Supplementary material for: International representation of authors, editors and research in neurology journals
Source: BMC Med Res Methodol. 2021 Mar 22;21:57. doi: 10.1186/s12874-021-01250-9 (PMC7983200; doi:10.1186/s12874-021-01250-9)
Supplement: Supplementary file 1 — Additional file 1 Table S1. First authorship (country). [file 12874_2021_1250_MOESM1_ESM.docx]

# Additional file 1

**Title**International representation of authors, editors and research in neurology journals
 **Authors
Name:** Teodora Bojanic
**Affiliation 1:** Faculty of Medicine, University of New South Wales, Sydney, New South Wales, Australia

**Email:** [t.bojanic@student.unsw.edu.au](mailto:t.bojanic@student.unsw.edu.au)

**ORCiD:** 0000-0003-3665-1764

**Name:** Aidan Christopher Tan

**Affiliation 1:** School of Medicine, Western Sydney University, Sydney, New South Wales, Australia

**Affiliation 2:** South Western Sydney Clinical School, University of New South Wales, Sydney, New South Wales, Australia

**Email:** A.Tan2@westernsydney.edu.au

**ORCiD:** 0000-0003-0354-4006

**Correspondence to:** Aidan Christopher Tan

**Postal address:** School of Medicine, Western Sydney University, Locked Bag 1797, Penrith NSW 2751, Australia **Contact e-mail:** A.Tan2@westernsydney.edu.au

**Telephone number:** 1300 897 669

**Article type:** Research article

**Word count:** 2348

## Table S1

| **Table S1. First authorship (country)** | | |
| --- | --- | --- |
|  | **2010** (n=729) | **2019**  (n=647) |
| United States of America (USA) | 220 (30%) | 198 (31%) |
| United Kingdom | 113 (16%) | 84 (13%) |
| Germany | 77 (11%) | 73 (11%) |
| Netherlands | 43 (6%) | 35 (5%) |
| France | 40 (5%) | 31 (5%) |
| Canada | 28 (4%) | 34 (5%) |
| Italy | 28 (4%) | 30 (5%) |
| Australia | 33 (5%) | 22 (3%) |
| Japan | 27 (4%) | 18 (3%) |
| Switzerland | 19 (3%) | 19 (3%) |
| Sweden | 16 (2%) | 13 (2%) |
| China * | 8 (1%) | 17 (3%) |
| Spain | 13 (2%) | 9 (1%) |
| Denmark | 6 (1%) | 16 (2%) |
| Belgium | 7 (1%) | 11 (2%) |
| Korea, Republic of (South Korea) | 4 (1%) | 9 (1%) |
| Finland | 7 (1%) | 4 (1%) |
| Austria | 6 (1%) | 4 (1%) |
| Israel and the Occupied Territories | 5 (1%) | 2 (0%) |
| Ireland, Republic of | 4 (1%) | 1 (0%) |
| New Zealand | 3 (0%) | 2 (0%) |
| Argentina | 4 (1%) | 0 (0%) |
| Taiwan | 2 (0%) | 2 (0%) |
| Portugal | 2 (0%) | 2 (0%) |
| Hungary | 3 (0%) | 0 (0%) |
| Poland | 2 (0%) | 0 (0%) |
| Brazil * | 0 (0%) | 2 (0%) |
| Singapore | 1 (0%) | 1 (0%) |
| Norway | 1 (0%) | 1 (0%) |
| Latvia | 1 (0%) | 1 (0%) |
| Malaysia | 1 (0%) | 1 (0%) |
| Turkey | 1 (0%) | 0 (0%) |
| Bangladesh * | 1 (0%) | 0 (0%) |
| Cyprus | 0 (0%) | 1 (0%) |
| Mexico * | 1 (0%) | 0 (0%) |
| Lebanon | 0 (0%) | 1 (0%) |
| South Africa * | 1 (0%) | 0 (0%) |
| Chile | 0 (0%) | 1 (0%) |
| Czech Republic | 0 (0%) | 1 (0%) |
| Russian Federation | 1 (0%) | 0 (0%) |
| Greece | 0 (0%) | 1 (0%) |
| * Developing country |  |  |
