# Supplementary material for: International representation of authors, editors and research in neurology journals
Source: BMC Med Res Methodol. 2021 Mar 22;21:57. doi: 10.1186/s12874-021-01250-9 (PMC7983200; doi:10.1186/s12874-021-01250-9)
Supplement: Supplementary file 2 — Additional file 2 Table S2. Origin of data. [file 12874_2021_1250_MOESM2_ESM.docx]

Additional file 2

**Title**International representation of authors, editors and research in neurology journals
 **Authors
Name:** Teodora Bojanic
**Affiliation 1:** Faculty of Medicine, University of New South Wales, Sydney, New South Wales, Australia

**Email:** [t.bojanic@student.unsw.edu.au](mailto:t.bojanic@student.unsw.edu.au)

**ORCiD:** 0000-0003-3665-1764

**Name:** Aidan Christopher Tan

**Affiliation 1:** School of Medicine, Western Sydney University, Sydney, New South Wales, Australia

**Affiliation 2:** South Western Sydney Clinical School, University of New South Wales, Sydney, New South Wales, Australia

**Email:** A.Tan2@westernsydney.edu.au

**ORCiD:** 0000-0003-0354-4006

**Correspondence to:** Aidan Christopher Tan

**Postal address:** School of Medicine, Western Sydney University, Locked Bag 1797, Penrith NSW 2751, Australia **Contact e-mail:** A.Tan2@westernsydney.edu.au

**Telephone number:** 1300 897 669

**Article type:** Research article

**Word count:** 2348

## Table S2

| **Table S2. Origin of data** | | |
| --- | --- | --- |
|  | **2010** (n=729) | **2019**  (n=647) |
| United States of America (USA) | 178 (24%) | 163 (25%) |
| International | 140 (19%) | 110 (17%) |
| United Kingdom | 88 (12%) | 69 (11%) |
| Germany | 64 (9%) | 61 (9%) |
| Netherlands | 34 (5%) | 32 (5%) |
| France | 32 (4%) | 30 (5%) |
| Canada | 23 (3%) | 25 (4%) |
| Japan | 24 (3%) | 18 (3%) |
| Italy | 17 (2%) | 23 (4%) |
| Australia | 24 (3%) | 14 (2%) |
| Switzerland | 17 (2%) | 12 (2%) |
| China * | 8 (1%) | 18 (3%) |
| Sweden | 11 (2%) | 10 (2%) |
| Spain | 11 (2%) | 9 (1%) |
| Denmark | 5 (1%) | 12 (2%) |
| Korea, Republic of (South Korea) | 4 (1%) | 9 (1%) |
| Belgium | 4 (1%) | 8 (1%) |
| Finland | 5 (1%) | 5 (1%) |
| Israel and the Occupied Territories | 5 (1%) | 3 (0%) |
| Austria | 3 (0%) | 3 (0%) |
| Taiwan | 2 (0%) | 2 (0%) |
| Hungary | 3 (0%) | 0 (0%) |
| Norway | 2 (0%) | 1 (0%) |
| New Zealand | 2 (0%) | 1 (0%) |
| Poland | 2 (0%) | 0 (0%) |
| Ireland, Republic of | 1 (0%) | 1 (0%) |
| Bangladesh * | 2 (0%) | 0 (0%) |
| Malawi * | 2 (0%) | 0 (0%) |
| Russian Federation | 1 (0%) | 1 (0%) |
| Argentina | 2 (0%) | 0 (0%) |
| Turkey | 1 (0%) | 0 (0%) |
| Bulgaria | 1 (0%) | 0 (0%) |
| Oman | 1 (0%) | 0 (0%) |
| Pakistan * | 0 (0%) | 1 (0%) |
| Colombia * | 1 (0%) | 0 (0%) |
| Philippines * | 1 (0%) | 0 (0%) |
| Cyprus | 0 (0%) | 1 (0%) |
| Brazil * | 0 (0%) | 1 (0%) |
| Vietnam * | 0 (0%) | 1 (0%) |
| Portugal | 0 (0%) | 1 (0%) |
| Mexico * | 1 (0%) | 0 (0%) |
| Romania | 1 (0%) | 0 (0%) |
| Tanzania | 1 (0%) | 0 (0%) |
| Latvia | 1 (0%) | 0 (0%) |
| United Arab Emirates | 1 (0%) | 0 (0%) |
| Saudi Arabia | 1 (0%) | 0 (0%) |
| Czech Republic | 0 (0%) | 1 (0%) |
| Singapore | 1 (0%) | 0 (0%) |
| South Africa * | 1 (0%) | 0 (0%) |
| Lebanon | 0 (0%) | 1 (0%) |
| * Developing country |  |  |
